# Fluid Extract Gelseminum

**Published:** 1874-03

**Authors:** 


Fluid Extract Gelseminum.—Dr. J. C. Howard, of Otter Creek, Florida, is making a fluid extract of gelseminum very superior to any now offered in the market. He informs us that it is tripple the strength of Tilden's—5 drops being equal to 15, the adult dose. The purity of Dr. Howard's preparation cannot be questioned, as he personally gathers the gelseminum and prepares the extract. Physicians wishing a pure and reliable extract can now secure one, by applying to Dr. Howard.

